# Significance of nucleophosmin1 (NPM1) gene mutation status on acute myeloid leukaemia patients with normal karyotype in South India

**DOI:** 10.1186/1755-8166-7-S1-P71

**Published:** 2014-01-21

**Authors:** R Sureshkumar, S Santhi, V Sangeetha, N Geetha, S Hariharan

**Affiliations:** 1 Division of Cancer Research, Regional Cancer Centre, Thiruvananthapuram, Kerala, India; 2 Division of Medical Oncology, Regional Cancer Centre, Thiruvananthapuram, Kerala, India

## Background

Acute myeloid leukaemia (AML) is a heterogeneous group of haematological malignancy. In spite of recurrent chromosomal abnormalities present in a significant proportion of AML patients, more than 50% of the patients have a normal karyotype (NK-AML) and lack reliable molecular markers. NPM1 has been recently characterized as the most frequently mutated gene in AML Patients. The objective of the present study was to profile the karyotype and to assess the types of NPM1 gene mutations in AML patients in south India.

## Subjects and methods

A total of 200 de novo AML patients were investigated in this study. Cytogenetic G- banding analysis, Fluorescence *in situ* hybridization was performed with standard methods. Mutation screening of NPM1 mutation hotspot exon12 performed in DNA by PCR-SSCP and suspected samples were sequenced.

## Results

Among 200 de novo AML patients 119 showed normal karyotype. Fourty out of two hundred patients showed mutations in NPM1 exon12. Most of the NPM1 mutation were detected in NK-AML (37/40) (92.5%).Three of them showed abnormal karyotype. The highest incidence of mutation was detected in AML-M1 with 47.5% followed by M5, M4, M2 and M3 .Predominant type of mutation was a type A mutation (c.860-863dupTCTG) (75%). Other type of mutations includes tetranucleotide insertion of TGCA (10%), CTGC (10%), GTCA (2.5%) and GTAG (2.5%). All the mutated cases were heterozygous in nature retaining wild type allele and have a distinct sequence in the protein c-terminal.

## Conclusion

Presence of 31% mutation in the NK-AML in the present study indicates that it is the most prevalent mutation in NK-AML patients in south India. NPM1 mutations are associated with good prognosis in AML. So predominance of NPM1 mutation in the AML especially in NK-AML patients suggests that it could be used as a reliable molecular marker for those patients.

